# A Qualitative Study of Antibiotic Use Practices in Intensive Small-Scale Farming in Urban and Peri-Urban Blantyre, Malawi: Implications for Antimicrobial Resistance

**DOI:** 10.3389/fvets.2022.876513

**Published:** 2022-05-24

**Authors:** John Mankhomwa, Rachel Tolhurst, Eunice M'biya, Ibrahim Chikowe, Pemphero Banda, Jimmy Mussa, Henry Mwasikakata, Victoria Simpson, Nicholas Feasey, Eleanor E. MacPherson

**Affiliations:** ^1^Malawi-Liverpool-Wellcome Trust Clinical Research Programme, Queen Elizabeth Central Hospital, Blantyre, Malawi; ^2^Liverpool School of Tropical Medicine, Pembroke Place, Liverpool, United Kingdom; ^3^Pharmacy Department, Kamuzu University of Health Sciences (KUHeS) Formerly College of Medicine, University of Malawi, Blantyre, Malawi

**Keywords:** antibiotic use, antimicrobial resistance, farming, poultry, Malawi, global south

## Abstract

The routine use of antimicrobials in meat production has been identified as a driver of antimicrobial resistance (AMR) in both animals and humans. Significant knowledge gaps exist on antibiotic use practices in farming, particularly in sub-Saharan Africa. This paper sought to generate in-depth understanding of household antibiotic use practices in food animals in urban- and peri-urban Blantyre. We used a qualitative research methodology focusing on households that kept scavenging animals and those engaged in small-scale intensive farming of food animals. Methods used were: medicine-use surveys with 130 conducted with a range of households; in-depth interviews (32) with a range of participants including farmers, community based veterinary health workers and veterinary shop workers; and stakeholder interviews (17) with policy makers, regulators, and academics. Six months of ethnographic fieldwork was also undertaken, with households engaged in farming, veterinary officers and veterinary stores. Our findings suggest antibiotic use in animals was more common in households that used small-scale intensive farming techniques, but rare in households that did not. For farmers engaged in small-scale intensive farming, antibiotics were often considered vital to remain solvent in a precarious economic and social environment, with limited access to veterinary services. A complex regulatory framework governed the import, prescription, and administration of antibiotics. Veterinary stores provided easy access to antibiotics, including colistin, an antibiotic on the WHO's critically important antibiotics for human health. Our work suggests that the high dependence on antibiotics for small-scale intensive farming may contribute to the growth of drug resistant infections in Malawi. The socio-economic drivers of antibiotic use mean that interventions need to take a holistic approach to address the high dependence on antibiotics. Key interventions could include improving farmers' access to affordable veterinary services, providing information about appropriate antibiotic use including withdrawal periods and feed supplementation, as well as improvements in regulation (nationally and internationally) and enforcement of current regulations. Taken together these approaches could lead to antibiotic use being optimised in feed animals.

## Introduction

Antimicrobial resistance (AMR) is considered one of the most pressing public health challenges facing humanity (1). Academic and policy work suggest that without concerted action, deaths from AMR could rise to 10 million per year, with the majority of these deaths occurring in Low and Middle income countries (LMICs) (2, 3). AMR is a complex, multifaceted problem that threatens both human and animal health (4). Recent global initiatives, including the World Health Organization (WHO) Global Action Plan on Antimicrobial Resistance, have been developed to galvanise a global collective response to AMR (5). The emergence and circulation of AMR-bacteria between animals, humans and the environment through sewage, water, and the food chain are considered to be critical points for the spread of AMR (6, 7). Recent global initiatives have therefore emphasised the need to address AMR through a One Health framework explicitly calling for multisector action (5, 8) but interventions that operationalise this approach have been slow to materialise (9).

Since the 1960s, routine use of antimicrobials in meat production has reduced the cost of food and allowed animals to be kept in smaller, more confined spaces, enabling significant gains in productivity, efficiency, yield and profit (10). Of the total global consumption of antimicrobials, an estimated 73% are used in animals raised for human consumption which we refer in this paper as food animals (11). In 2010, it was estimated that 63,151 tonnes of antimicrobials were used in food animals, with a projected rise to 105,596 tonnes by 2030, an increase of approximately 69% (11). Two-thirds of this increase is attributed to the growing number of animals raised for human consumption with a third of the increase due to predicted shifts in farming techniques, with intensive farming becoming more prevalent (12). India, China, Brazil and South Africa are projected to have the greatest increase in antimicrobial consumption—up to 99% over the 20 year period, which is approximately seven times the projected population growth of these countries (11). The regular dosing of food animals with sub-therapeutic doses of antibiotics to promote growth and prevent disease has been identified as a potential driver of antimicrobial resistance in both human and animals and has been banned in the European Union since 2006 (7, 13, 14). Globally, there is a growing movement to curb the excessive use of antibiotics both prophylactically and to promote growth (15). Particularly for antibiotics such as colistin which is a last line or ‘reserve’ antibiotic considered of critical importance to human health (16). However, complex regulatory frameworks combined with limited resources to enforce regulation makes reducing antibiotic use challenging (17).

In LMICs, there has been a substantial growth in small-scale intensive animal farming. Farmers engaged in intensive farming, keep high-yield breeds that rely entirely on farmers for their feed and are kept in small enclosures (18). The introduction of these animal husbandry practices has increased production but antibiotics are an integral part of farming these animals (11, 19). In LMICs, access to veterinary and diagnostic services are limited, yet infectious disease rates are high meaning that antibiotics are often essential (20). Antibiotic use in small scale intensive farming can be conceptualised as a form of infrastructure which enables farmers to overcome fractured animal health systems to make a profit and secure food (21). The infrastructural role of antibiotics within food animal production in low-and-middle-income countries mean that the regulation may have unintended consequences. Hedman et al. (22) noted that the ban on antibiotic growth promoters in poultry production in South Africa would have a negative short to medium impact on the food security of humans in the entire Southern African Development Community (SADC) region (22).

As farming practices become more intensified in LMICs, with a greater integration of sub-therapeutic dosing in food animals, wide-reaching implications for AMR in both humans and animals are likely. However, given the potential for unintended consequences when reducing antibiotic use in food animals, there is a need to develop context-specific responses that reflect these challenges. Much of the research undertaken to date has explored middle-and upper middle-income countries and a significant research gap exists in low-income countries, particularly in Africa (23). In low-income contexts such as Malawi, where this study was undertaken, chickens were the most commonly intensely farmed animals reflecting wider trends across LMICs (24). In 2019, the poultry population of Malawi was more than 190 million, which was a 24.5% growth in numbers from the previous season suggesting growth in the sector (24). Farmers work in a context where food insecurity is widespread, there is multi-dimensional poverty, and constrained access to veterinary services including professional expertise. There has also been notable increase in drug resistant infections in both adults and children at the central referral hospital demonstrating that AMR is becoming a growing concern (25, 26).

There were significant geographical and economic inequalities that made accessing care for sick animals challenging (27). Community based veterinary health practitioners included: Veterinarians, veterinary officers, assistant veterinary officers, para-veterinarians, and veterinary scouts. Officially, Veterinarians and veterinary officers were responsible for providing veterinary services including prescribing antibiotics. Veterinary scouts and para-vets provided support with disease surveillance and prescribed over-the-counter medicines such as de-wormers. There were very few trained veterinarians in the country, as the programme was only established at Lilongwe University of Agriculture and Natural Resources (LUANAR) in 2012, with the first gradates completing the course in 2019.

This study was undertaken in Malawi and the research was guided by three key research objectives: (1) to identify the types of antibiotics being used in households for food animals farming in urban and peri-urban Blantyre; (2) to understand the ways antibiotics are being accessed and used by farmers in urban and peri-urban Blantyre; (3) to understand how the broader social, economic, and political environments are shaping use.

## Materials and Methods

### Study Design

This study used qualitative research methods to provide an in-depth understanding of how antibiotics were accessed and used by households who kept food animals in urban and peri-urban in Blantyre. Our methodology was informed by a One Health approach, with the design, sampling and analysis focused on drawing out the connection between humans and animals within selected households. In a research area where little work has been undertaken, exploring the use of antibiotics in small scale animal farming through qualitative research methods allowed us to capture the lived realities of farmers from their own perspectives (28). The study ran from March 2019 until April 2020. It was part of the Drivers of Resistance in Uganda and Malawi (DRUM) study; a multidisciplinary study that aimed at modelling the drivers of AMR in Malawi and Uganda.

### Study Setting

Blantyre District covers 228 km^2^ with densely crowded settlements and was estimated to have a population of nearly 1 million, making it the second largest city in Malawi, after the capital, Lilongwe (29). This study was situated in two distinct geographical areas (a peri-urban location) and Ndirande (an urban location) in Blantyre District of Malawi. This enabled us to compare farming in an urban informal settlement and a peri-urban context. Ndirande is a densely populated township characterised by poor living conditions and precarious livelihoods on the outskirts of the city. It was designed during colonial times to house indigenous Africans with extremely limited public services (30). In contrast, Chileka is located on a plain between the Shire Highlands and the Shire River. Chileka is considered peri-urban, and residents have significantly better access to land than Ndirande.

### Data Collection

The methods used included medicine-use survey, ethnographic fieldwork, key informant, and stakeholder interviews. The study team consisted of JM, EM and three research assistants JMu, HM and PNB. JM is a trained medical anthropologist, fluent in Chichewa and English who assumed both an insider role and outsider as he had not previously undertaken farming. He led the fieldwork with support from three research assistants. To ensure quality of the data collected, extensive training and support in data collection and analysis was delivered by EM.

## Recruitment and Sampling

### Medicine-Use Survey

We began the study by undertaking a purposively sampled survey to understand household antibiotic use practises for both humans and animals. We conducted 130 interviews in total, with households sampled based on the presence of livestock. In the two sites, Ndirande and Chileka, we sought to purposively sample 65 households in each site, ensuring we covered the whole geographical area and captured data on both subsistence farmers and those engaged in small-scale intensive farming. We used a “drug-bag” method which uses a series of pile sorting exercises to allow participants to identify which medicines they recognise, ever used, and frequently used. We also asked participants about where they accessed medicines. This method aided discussion and helped overcome linguistic barriers as the term antibiotic is a category of medicines that are not readily understood in Malawi (31). To assemble the drug bag, the research team visited formal and informal access points for veterinary and human antibiotics (31).

### Ethnographic Fieldwork

During the ethnographic fieldwork the team spent time observing farmers and veterinary officers (32). We sampled farmers engaged in small-scale intensive farming, reflecting the definition of the FAO, these high-yield breeds relied entirely on farmers for their feed, kept in small enclosures or within the farmer's house as opposed to subsistence farmers whose animals were scavenging or semi-scavenging (18). We identified these farmers through the medicine survey and from snowballing farmers. In total we followed 29 farmers in the two sites engaged in small scale intensive farming. The study team were participants as observers spending time within the households over a 6-month period, helping with small jobs and building a relationship with the farmers (32). Observations focused on understanding farming as a livelihood strategy, animal husbandry techniques and use of medicines paying careful attention to whether farmers used antibiotics in what ways and how they were accessed. We also spent time with community based veterinary officers, visiting farmers and understanding their day-to-day work activities. After each day, the researchers wrote up fieldnotes and shared reflections at weekly debriefing sessions.

### Structured Observations

To understand access to and dispensing of animal antibiotics we also conducted structured observations in veterinary stores in Blantyre. We considered five sites where farmers and veterinary officers bought medicines for animals, from which three were selected because they were the busiest and sold more medicines than the rest. We spent a month visiting each shop daily and a total of 230 interactions were observed over a 3-month period. During the interactions, notes were taken on the types of medications purchased, the types of animals requiring treatment and how they were dispensed. The team paid careful attention to the types of questions and conversation between the customers and those dispensing the medicines.

### In-depth Interviews

We conducted 59 qualitative interviews with a range of purposively selected participants (see Table 1). These included 29 in-depth interviews with farmers, 6 veterinary shop attendants and 7 veterinary officers all of whom had been included in the observations in the communities and veterinary shops. Finally, we conducted 17 key informant interviews with stakeholders sampling policy makers at district and national level, those employed by the regulator and academic working in animal health.

**Table 1:** Type of respondent sampled in the qualitative interviews.

**Type of Respondent**	**Male**	**Female**	**Total**
**Farmers**	14	15	29
Broilers (6)			
Layers (3)			
Mixed (6)			
Goats (7)			
Pigs (2)			
Mixed (5)			
**Veterinary prescribers and dispensers**	10	3	13
Government veterinary officers (4)			
veterinary store attendants (6)			
Private veterinary officers (3)			
**Stakeholders**	11	6	17
Academic institutions (2)			
Animal health representatives within national and local government (4)			
National stakeholders (regulatory and policy makers) (11)			
Total interviews	36	23	59

### Data Analysis

We analysed the data using thematic content analysis (28). All interviews were recorded, transcribed, and translated into English by the research team. Fieldnotes were collected from each medicine-use interview, in-depth interview, and observations. Data analysis began alongside data collection and as a team we used weekly debriefing meetings to reflect on initial findings and identify new avenues for further enquiry (33). We developed an initial thematic coding frame during the debriefing sessions. After the fieldwork was complete, a common thematic framework was developed and all transcripts and fieldnotes were imported into NVIVO 12 and coded line-by-line to group ideas and explain emergent phenomena.

## Results

We present our results around three themes: (1) Precarious livelihoods and unequal market structures in urban and peri-urban Blantyre, (2) Use of antibiotics and its drivers in small-scale intensive farming, (3) Complex governance structures and fragmented regulatory frameworks.

In the first theme, we document farmers' livelihood precarity and the challenges they faced in generating sufficient profit to remain solvent. In the second theme, we explore how tight economic margins, and limited access to veterinary services combined with open access to veterinary shops shaped antibiotic use for some farmers. In the final theme, we trace the historical evolution of relevant policies and explain how these underpin the fragmented regulatory framework. We also explore the wider governance challenges that shape current day access to and use of antibiotics.

## Precarious Livelihoods and Farming Practices in Food Animals in Urban and Peri-Urban Blantyre

### Animal Husbandry Practices Within Precarious Livelihoods

Famers were engaged in small-scale intensive farming in both urban Ndirande and peri urban Chileka, with distinct differences in patterns of farming between the two locations. In Ndirande, broiler and layer chickens were the most frequently farmed animals. Space was a significant challenge and animals were predominantly kept within the confines of the farmers houses. This limited the number of birds' farmers were able to keep. Flock sizes ranged between 70 and 150, with one farmer keeping 300 cages within the compound of her house. The by-laws of Blantyre City Council prohibited livestock from being kept within the city boundaries to prevent animal-to-human infections. Despite this directive, we found that livestock including pigs and cows were kept in Ndirande. However, farmers lived under the threat of eviction if they were caught. In Chileka, which is situated outside the city boundaries, a wider range of animals were farmed both intensively or kept as scavenging or semi-scavenging livestock, including cattle, goats, sheep, pigs, rabbits, and poultry. This was because farmers in Chileka had comparatively more land available to construct *kraals* (an animal enclosure built out of wood or metal to contain animals). Some households kept animals inside the house because of security concerns and for those engaged in small scale intensive farming, animals were closely monitored for disease.

For farmers in Chileka and Ndirande, engaging in small-scale intensive farming required significant capital outlay. Managing this capital was a constant pressure for the household. For intensive poultry farming, farmers were required to buy chicks, feed, and medicines. Broiler and layer farmers frequently entered and exited the business if they were unable to keep the business solvent, often if animals had died due to illness or pests. The uncertainties and challenges of staying afloat in the small-scale intensive poultry business was frequently discussed by farmers. This is articulated below:

*We started this type of farming after my husband quit his job, thus we decided to invest his gratuity in poultry farming. He was working at FINCA [a microfinance organisation] and after quitting we started animal farming which included pigs, broilers, and layers. However, now we are only keeping pigs and broilers. We stopped rearing layers after red ants spread in our Kraal and exterminated 200 chickens which were all we had, and this led into a huge setback, so we decided to just continue with broilers and pigs*. (IDI, Pig, and poultry farmer, Ndirande)

In the 130 households sampled animals kept included goats, pigs, local chickens, and imported chicken breeds (either layers or broilers). Chickens were the most frequently kept (both local and imported breeds). For most households, animals were kept inside for at least part of the day to protect them from theft, disease or due to lack of space to build a *kraal* or other permanent structure. Households keeping animals on a smaller scale usually kept a few goats or local chickens. They were often kept freely roaming in the wider locale during the day. Local chickens and goats represented a form of capital for the household that could be sold if or when the household needed money. Households farming more intensively kept broilers, layers, goats, and pigs in larger numbers often in a small space. The movements of these animals were more controlled, with them rarely, if ever, leaving the living space. Intensely farmed animals occupied a different role in household livelihoods. Income generated from these activities was often the primary source of income for the household, as narrated below by a broiler farmer:

*The main aim for starting Broiler farming was to have a reliable source of income. I wanted this to be the main source of income for my family, where we can easily get money and put food on the table because to live our lives, we need money, and you cannot have children and lack money to support them*. (IDI 2, Blantyre poultry farmers association)

Keeping broiler and layer chickens was the most labour intensive. Farming these chickens required farmers to have permanent ownership of the house and/or land to build structures suitable for keeping animals. Landlords would not permit large numbers of chickens to be kept inside their properties due to smell and mess. Farmers carefully monitored these animals giving water and feed mixes daily. For broilers, day old chicks would be kept for 5–6 weeks before slaughtering. Layer birds were kept for around 1 year, and required constant feeding, medicine and general care required to keep them alive. Keeping birds healthy in confined conditions was often challenging.

### Unequal Market Conditions

Feed shops, poultry-producing companies, and veterinary Shops were the main source of all farming-related products. Most farmers in the study entered the broiler and layer markets through purchasing day-old pre-vaccinated chicks or fully-grown laying chickens from the large poultry producers. The companies also provided loans to farmers to support entry into the trade. We found four large poultry producers who offered these services. Farmers had complex relationships with these companies because the companies sold not only chicks, but also fully grown broilers (live or in small parts) and eggs. They sold these in urban and peri-urban areas from vans, charging a lower price which undercut the business of local farmers. This unequal competition generates frustration and resentment, as narrated by a farmer below:

*So, what is happening is that the big companies like [names two poultry producers] and others who are able, take their chickens in pickups to where we sell ours and sell them at very cheap price. For example, I am selling my chickens at MK 3,500 [US$4] and they are selling theirs at MK 2,500 [U$3]. So, these are some of the things pulling us down, I do not know if the government allow them to make feed, selling chicks selling chickens but also eggs because all of these are challenges to small scale farmers, and it is hurting us. At the end we are not developing as farmers we are still where we were*. (IDI, Poultry farmer, Blantyre)

Whilst some farmers viewed their relationship with these poultry producing companies more positively, the use of loans secured against stock in the unequal market conditions described above presents substantial risk for small-scale farmers companies:

*They [one of the chicken producers] have this other program where they give people chickens and feed on loan, but I do not know the agreement. You pay a deposit they bring feed, those cages and the chickens and they visit them. In their agreement you are given chickens and feed on loan. So, it is like this week you are going to collect feed and you are paying the debts of the feed you collected last week. If you have failed to pay the debts, they take ownership of the chickens. If you finish paying the debts, then the ownership is yours. Despite paying the debt the partnership it's still there because you are still using their cages and you are told to buy feed from them*. (IDI, Poultry farmer, Ndirande)

Without the option of accessing loans, few farmers were able to enter or re-enter the trade after economic loses.

The companies also provided farmers free veterinary services, as they employed private veterinary officers. Trainings supported entry into the poultry industry and included good husbandry techniques, as can be seen below:

*Yes, I was trained in poultry farming, first I learnt it from my friends who were also keeping chickens, but I had to learn it formally when I attended a training on poultry production at [one of the big feed producers]*. (Blantyre Poultry farmer's Association, farmer IDI)

Overall, farmers faced significant challenges sustaining their livelihoods and supporting their families, particularly when buying and growing broiler chickens. The large poultry producers, while providing some access to veterinary services and credit, also dominated the market by selling chickens for consumption in vans at a cheaper price which undercut farmers.

## Antibiotic Use and Its Drivers of Antibiotic in Small-Scale Intensive Farming

### Recognition and Reported Use of Antibiotics Within Households

**Figure 1:**
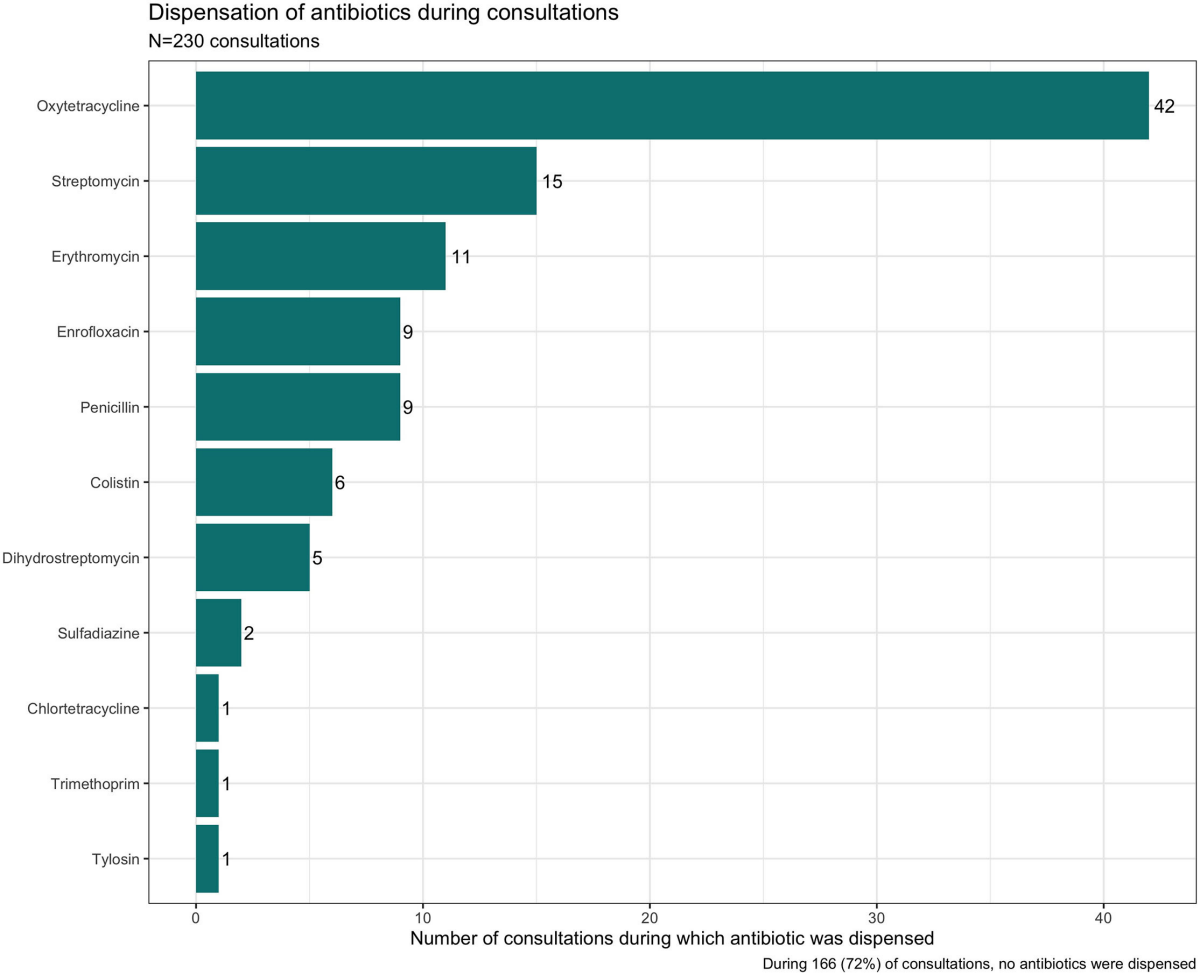
Recognition of animal antibiotics in Malawi.

**Figure 2:**
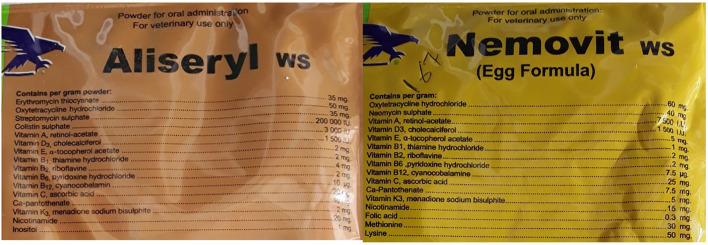
Recognition of human antibiotics in Malawi.

**Figure 3:**
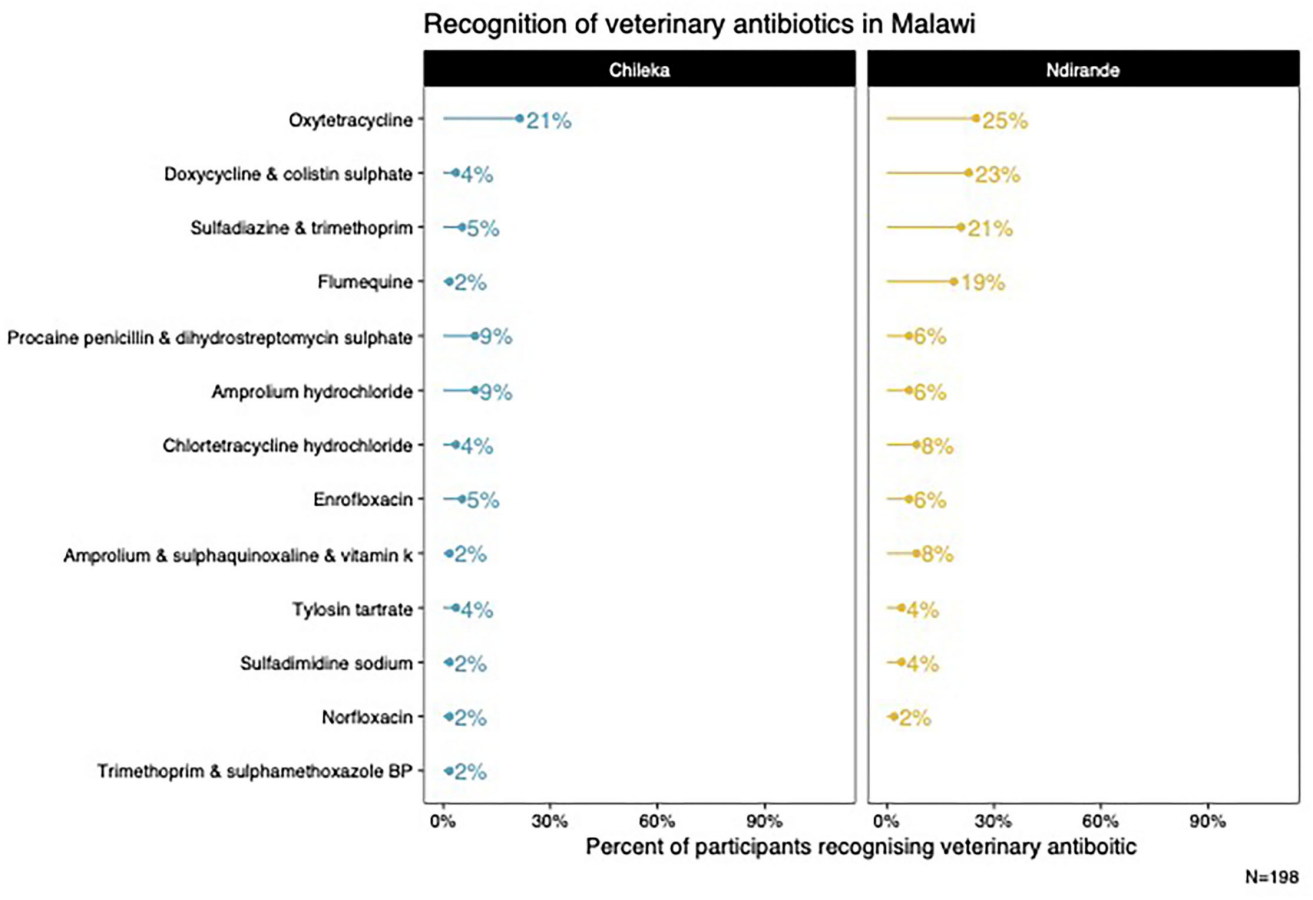
Frequently used animal antibiotics Malawi.

**Figure 4:**
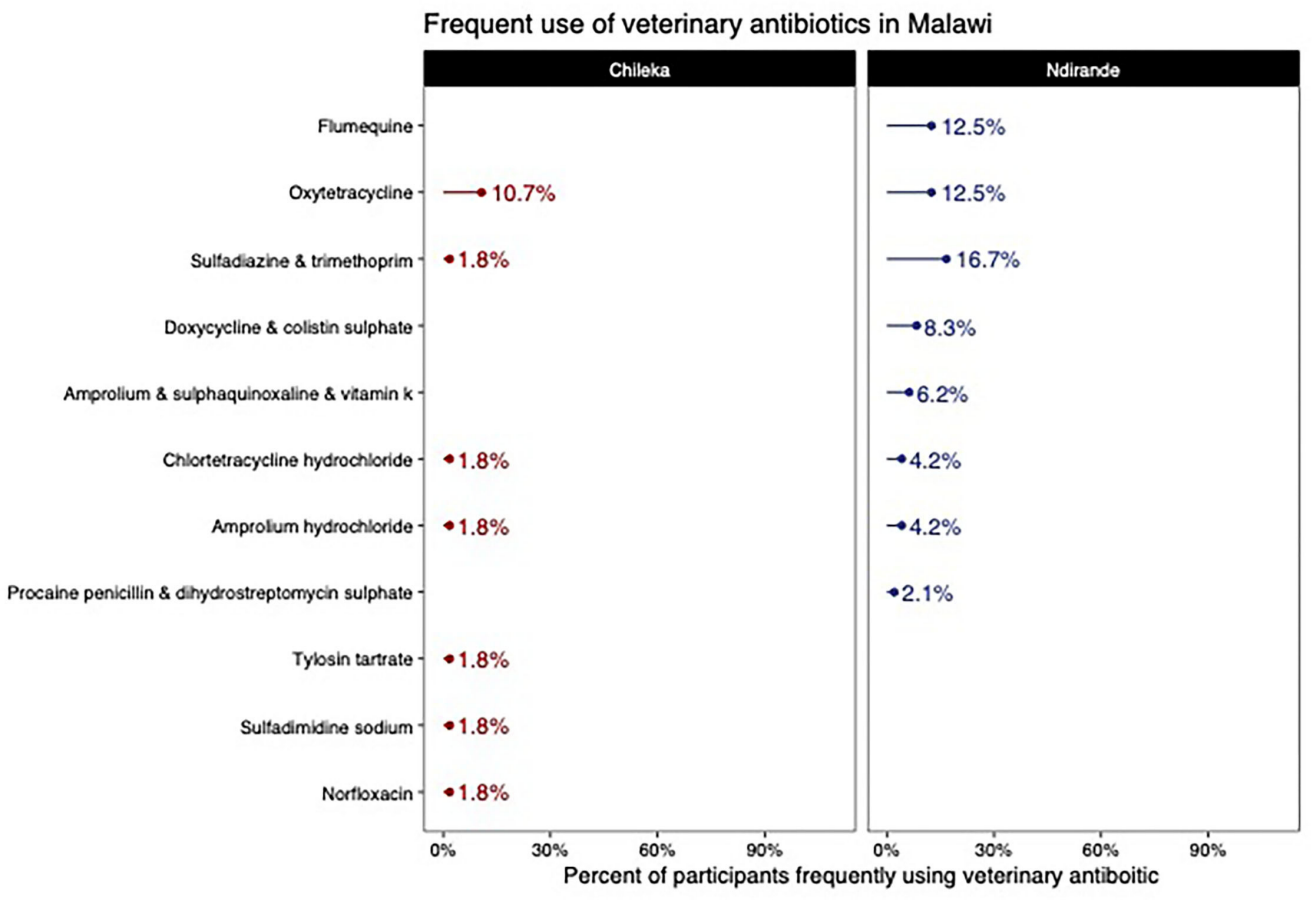
Frequently used human antibiotics Malawi.

**Figure 5:**
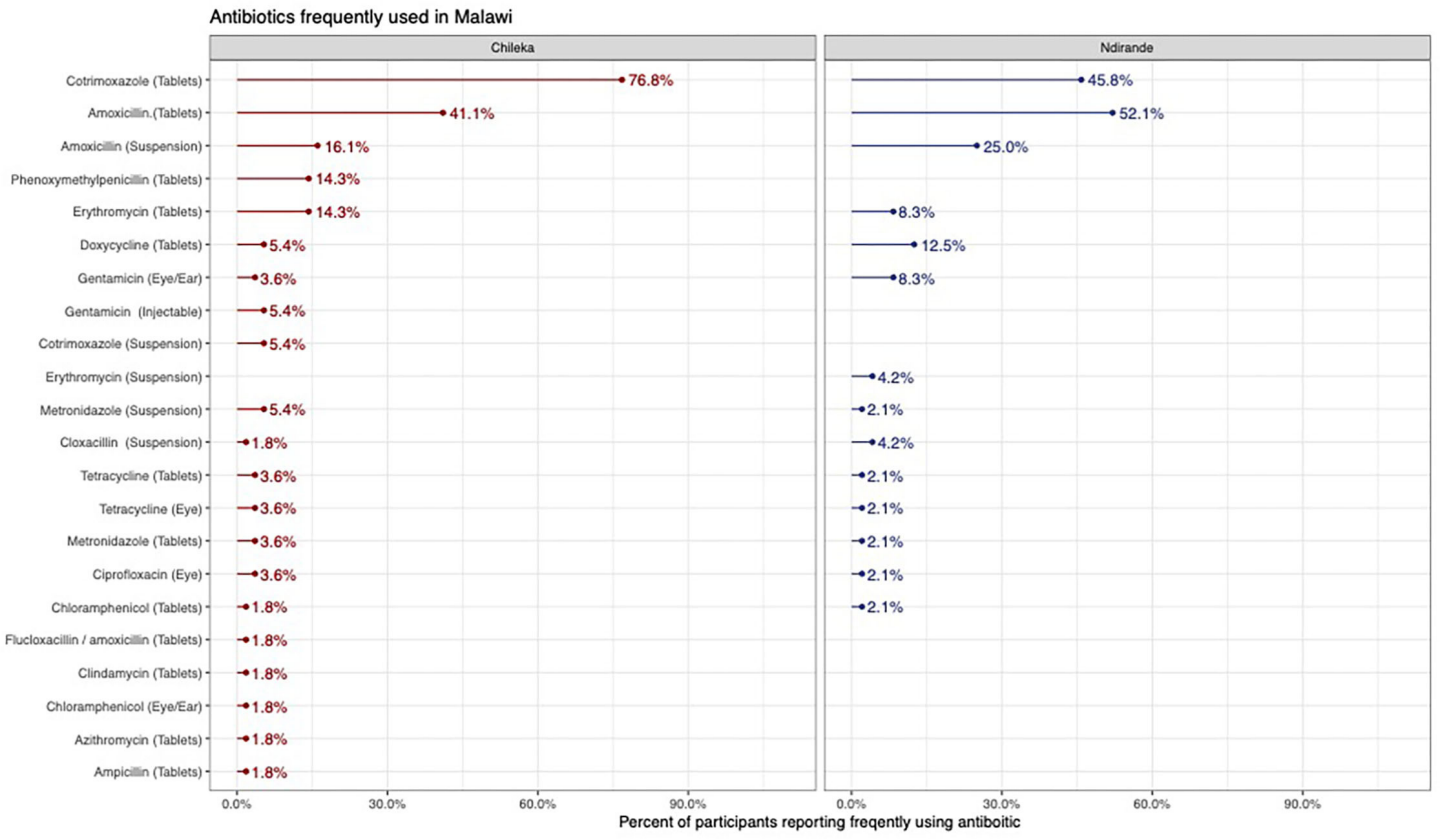
Antibiotics Dispensed in the veterinary shops and interactions observed.

From the medicine survey we undertook with 130 households across the Ndirande and Chileka to understand the types of animal and human antibiotics used within the household. In Figure 1, we present the most frequently recognised animal antibiotics in both sites. Oxytetracycline was the most frequently recognised in both sites. However, there was a clear difference between Chileka and Ndirande, with people in Ndirande more frequently recognising animal antibiotics than those living in Chileka. In Figure 2, we present the most frequently recognised antibiotics in the two communities, and there is considerably higher recognition of human than animal antibiotics. In Figure 3, we present the most frequently used animal antibiotics within the households. In Figure 4, we present frequently used human antibiotics, with amoxicillin and cotrimoxazole being the most recognised. Mirroring the findings from the recognition data, we see that human antibiotics were much more frequently used. In Figure 5, we present the household recognition data. In Ndirande, antibiotics were more frequently used antibiotics than in Chileka. In both sites, we found a marked difference between those that kept scavenging or semi-scavenging animals compared to those engaged in small-scale intensive farming, with households engaged in small-scale intensive farming far more likely to recognise and use antibiotics than those who did not.

### Types of Antibiotics Dispensed From the Veterinary Shops

The veterinary shops were an important source and of antibiotics for both community-based animal health practitioners (including veterinary officers) and farmers. These shops were staffed by shop attendants and a veterinary officer as per the regulation. Dispensing was made either based on symptoms described by the customers or requests for specific drug. During our observations, we found the environment to be very permissive, and customers were never refused sales. If the farmer was unfamiliar with the medication they required, then the shop attendant would dispense based on their knowledge of the medicines in stock.

**Figure 6:**
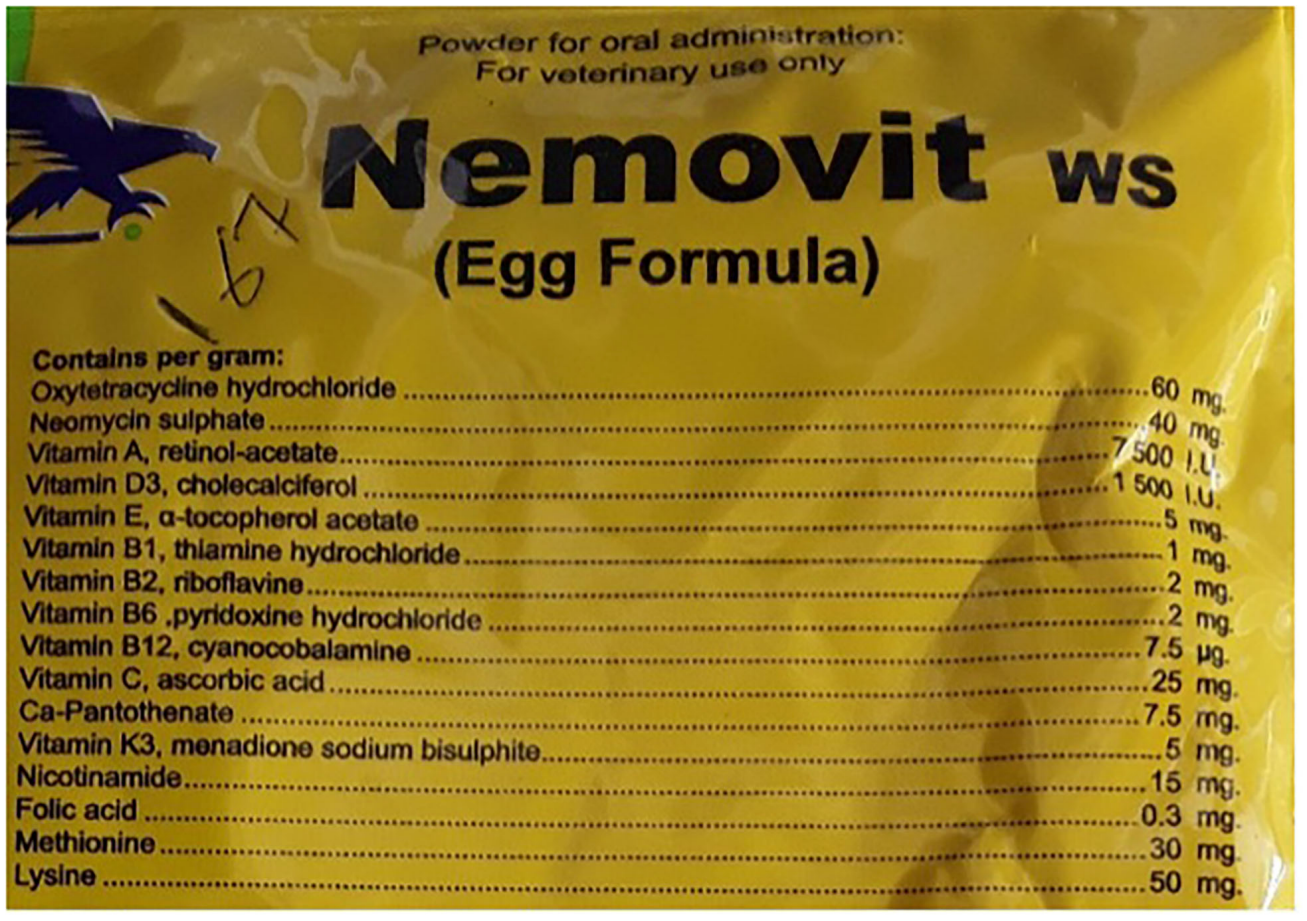
Examples of antibiotics dispensed.

Most of the consultations (72%) did not end in an antibiotic being dispensed. Several factors may have shaped this. During the consultations other medicines including anti-parasitic drugs and vaccines were dispensed without antibiotics. The veterinary antibiotics were expensive so when customers enquired, they did not always purchase the medicines. Finally, the shops were also an important source of information where farmers could go to gain information on nutrition and keeping animals healthy. As can be seen in Figure 5, oxytetracycline was the most frequently dispensed antibiotic but often in combination. See Figure 6, for an example antibiotic mix—*Aliseryl WS* contained erythromycin, oxytetracycline, colistin and vitamins. Despite, colistin being banned in animal use in Malawi, it was dispensed. When we probed shop attendants and veterinary officers, they were unaware of this regulation. During our observations we did not find vitamin mixes (used to ensure good growth) without antibiotics, meaning animals were regularly given low doses of multiple antibiotics.

Figure 6 provides an example of the vitamin mix dispensed.

### Livelihood Imperatives for Antibiotic Use

We identified a complex set of drivers of antibiotic (non)use. We use the term (non)use because there were times when antibiotics may have been needed but were not used.

The cost of animal antibiotics was a significant factor in shaping farmers decisions around use. Animal antibiotics were considerably more expensive than human antibiotics. For scavenging and semi- scavenging animals, the use of animal antibiotics was rare, for small-scale intensive farmers, use was more frequent but cost also prohibited use. Antibiotics for chickens, such as *Nemovit* or *Aliseryl WS* which included vitamins, cost approximately MK 3500-4500 (~$4.50–$5.50) per packet. Pen-Strep—an injectable often administered to livestock—cost MK 7000–MK 8000 (~$7–8) at the veterinary store. In contrast cotrimoxazole (a broad-spectrum antibiotic for human use) could be purchased on the informal market for MK 300-500 ($0.30–0.50) for 10 tablets. Some households reported using cotrimoxazole for their animals (scavenging and small-scale intensive), particularly if their chickens were sick:

“*I give Bactrim [cotrimoxazole] to my chickens. When I need it, I go and buy medicines in town. I dilute in water and give them to chickens to drink.”* (Medicine interviews, respondent 97, male poultry farmer Ndirande)

During the interviews, stakeholders reflected on the ways animal husbandry techniques for small-scale intensive farmers shaped the burden of disease in these animals. This related to the confinement combined with limited biosecurity that meant animals fell sick more frequently:

*You see a marked difference in the disease burden when animals are kept in proximity and intensely farmed. Things you do not see when animals are kept freer, local chickens are just not getting these diseases.… For those farming intensely, they have their own drug bags, they may call you to come and see say a sick pig, but by the time you get there, they have already given the antibiotics. But there is no biosecurity, disease burden is high…. if they were not able to keep the drugs, they are abusing them of course, but a lot of the pigs would be dying, without them*. (Stakeholder interview national policy representative)

In the quote, the stakeholder reflects, both on the animal husbandry techniques, limited biosecurity, and dependence on antibiotics. Reflecting the complex dilemmas that farmers faced when it comes to using antibiotics. A further challenge faced by some small-scale intensive poultry farmers, was the cost of cleaning products. For some they were unaffordable, and these households relied on deep cleaning and changing rooms after they had sold the poultry (up to 6 weeks at a time). This reflects the precarious economic position some small-scale intensive farmers faced.

Ensuring that animals remained healthy was a key concern for small-scale intensive farmers and they carefully watched their animals for any changes in appetite or behaviour. Some farmers used antibiotics to avoid them becoming sick. In this quote below a farmer describes how he uses injectable antibiotics to prevent sickness:

*I can say that I frequently inject them with Hi-Tet [Oxytetracycline Long Acting] because it is a broad spectrum and treats a lot of illnesses. I administer it to the goats before they fall ill because it helps in preventing them from falling ill*. (IDI, Male Goat farmer, Ndirande)

The economic imperative for farmers was always to sell live animals even if they were sick to remain economically viable. Farmers were still able to sell their animals if they died naturally, but it meant a significant reduction in money realised and posed a significant risk of insolvency. As noted below:

*We sell our chickens to various people who include people owning restaurants around the Ndirande market area and French fries vendors, they either buy live chickens or dead ones, for the dead ones normally the price is reduced so that I should not lose out completely but rather make a small profit out of it because this is all my money invested here*. (IDI, Male Broiler farmer, Ndirande)

Farmers faced a short window to decide whether to use antibiotics to treat their animal's sickness or sell them to ensure they are not wiped out financially. If farmers had antibiotics at home or were able to borrow from a friend, they would dose to see if there was any improvement. If farmers had to pay for antibiotics, they would carefully weigh up the economic costs and slaughter quickly if they lacked the funds to treat. In these times, it is likely that animals did require treatment, but famers precarious livelihoods shaped this.

*I can give you an example, when two of my chickens stopped eating and were showing signs that they were going to die so I killed them and sold them to some of my customers*. (Medicine-use interviews, broiler farmer, Ndirande)

Where animals given antibiotics showed little improvement they would be quickly slaughtered. During the fieldwork, farmers never discussed the withdrawal period and when medicine were dispensed at the veterinary shops there was no discussion or information given to the farmers on this.

### Veterinary Care Access and Antibiotic Use

Access to community based veterinary care also shaped antibiotic use practises for small-scale intensive farmers. While the Malawian regulatory framework states that farmers should consult veterinary officers to access antibiotics in practise this was rarely enforced.

Farmers accessed information and medicines (including vitamins, vaccinations, and antibiotics) from a range of sources. These included lead farmers, veterinary shops, or community-based providers such government and private veterinary officers, para-veterinarians, and assistant veterinary officers. There were several barriers to farmers accessing veterinary officers. To visit the farmer veterinary officers charged, fees for consultation, medicines, and transport. In Chileka, three veterinary officers served a large geographical area (14.5 Km^2^). In Ndirande, there was no government veterinary officer, meaning that if farmers wanted them to visit their animals, they had to travel further. Relationships between farmers and the community-based veterinary practitioner were complex. Farmers complained about how challenging it could be to access prompt veterinary services, particularly veterinary officers who were limited in numbers. Given the tight timeframes for farmers to decide whether to treat, sell or slaughter their animal, the slowness of response drove farmers to seek veterinary advice from other farmers or buy antibiotics at the veterinary shops and treat. Farmers described their lack of trust in veterinary officers, who they felt were not transparent in their practises. During our observations when veterinary officers administered antibiotics, particularly injections, they would hide the bottle. This practise may in part, be about enclaving knowledge about the medicines so farmers could not simply go and buy the medicines without the veterinary officers.

In contrast for some farmers, veterinary officers were the voice of authority and provided an important gateway to good livestock production practises. If farmers were struggling to access funds, they could also get medicines on credit from the veterinary officer. This was noted by a goat farmer in Chileka when asked about the knowledge of medicine administered to their animals:

*I have never asked but most of the times when he [veterinary officer] comes and I tell him that my goat is not feeding he just says I will give it some medicine it will start feeding soon and he then administers an injection. One was given an injection that was driven deep in the flesh another, one was given an injection in the skin I was not interested to ask what kind medicine it was, but now when I call him again, I will ask him what kind of medicine it is*. (IDI, Male goat farmer, Chileka)

Farmers did not always use biomedicines to treat animals, instead choosing herbal medicines such as aloe vera, the bark of mango trees and Moringa tree leaves. This was a common practices among poorer farmers who had less income available to buy animal medicines but was also used by famers who felt antibiotics had not worked treating recurring illnesses. The use of herbal medicines was more common in Chileka than in Ndirande.

### Feed and Antibiotics

Feed was also an entry point for antibiotics into the food chain. Feed companies which were predominately located in central Blantyre manufactured and sold feed to farmers. They sold specific feed that catered for the broiler and layer market, with feed for each stage of the 6-week process in broilers. While the bags of feed did not explicitly state which antibiotics they contained, they did use the terms “growth promoter” and “coccidiosis preventers” suggesting that they did contain antimicrobials. Over the course of the study, we found that these terms were removed from the labels. As one stakeholder reflected, the Central Veterinary Laboratory did not have the funds and equipment to analyse feed samples to determine what compounds were present.

When we explored with farmers what they thought was present in the feed, most knew it had some important properties to keep the animals growing well but there was a lack of understanding on what these compounds consisted of:

*What I have observed is that, when one is feeding the chickens feed from the shops, within a short period of time the chickens grow bigger, [so I believe there is something that is put in the feed to enable the chickens grow quicker]- that's why their feed is expensive*. (IDI broiler farmer, Ndirande)

## Governance Structures and Regulatory Frameworks

### Regulation for Prescribing Antibiotics for Animals

The first policy statement of the Department of Animal Health and Livestock Development (DAHLD) was published in 1952 and was reviewed in 1972 and 1988. The policy statement focused on improvements in livestock production through disease control. Building upon this, the MoA developed the *Agricultural and Livestock Development Strategy and Action Plan* (ALDSAP) in 1995, the *Ministry of Agriculture Strategic Plan* in 2002, and *Road Map on Agricultural Development in Malawi* in 2005 as operational tools. The Ministry's strategic objective recognises the importance of the livestock industry in food security and poverty reduction. Veterinary shops and DAHLD are responsible for selling and distribution of antibiotic medicines. According to the MoA, through the Department of animal health and livestock Development (DAHLD) the Veterinary and Para-Veterinary Practitioners Act of 2001 places veterinary officers at the centre of antibiotic prescription and administration. This framework was described by a key informant:

*The government has put in place a law that controls the activities that relate to veterinary medicines in our sector. For instance, all antibiotics are supposed to be administered by qualified veterinary officers only, if antibiotics usage is not regulated animals may develop resistance to them. Thus, field workers who have no veterinary background are only allowed to administer medicines which are not sensitive like de-wormers or medicines that treat or dress wounds and not antibiotics because they are quite sensitive* (Key informant interview national policy representative).

As described in the above theme, the extremely limited number of veterinary officers available constrained timely and affordable access by farmers. This led to a wider range of actors being involved in the administration of antibiotics and little adherence to the guidelines. National policy representatives reflected on how the limited number of veterinary officers available shaped the way surveillance activities were implemented:

*Unfortunately, our role is to make sure that; number one there is a veterinary officer there who will be assisting people accordingly, two even though we don't do it systematically we go and check expiry dates for the medicines being sold in the veterinary shops, but I tell you last year we did this exercise once and we haven't done it this year so far due to lack of funding for the exercise*. (IDI Key informant interview national policy representative)

### Registration and Importation of Antibiotics

Malawi does not have a large domestic pharmaceutical industry and we found no medicines aimed at the veterinary or human market manufactured in country. Table 2 below shows the countries where the medicines were manufactured, the most common being the Netherlands and Tanzania.

**Table 2:** Country of manufacture for animal medicines found for sale in Malawi.

**Country**	**Number found**
Netherlands	30
Tanzania	23
UK	12
Kenya	11
USA	4
France	2

All medicines, including antibiotics used in farming were imported through two routes. Firstly, the government imported medicines, often in partnership with NGOs working on animal health, to treat disease outbreaks and for mass vaccination campaigns such as rabies. Secondly, medicines were imported through licences issued by the Ministry of Trade, Commerce, and Industries (MTCI). The Pharmacy and Medicines Regulatory Authority (PMRA) in conjunction with the Department of Animal Health and Livestock Development (DAHLD) provide a list of recommended medicines that can be imported into the country and the licences provided need to reflect this list. However, significant logistical and financial barriers meant implementing these procedures was challenging particularly as Malawi's borders were porous. This was explained by the veterinary officer below:

“*The other thing is some antibiotics come into the country unregistered by Medicines and Poisons board. Now I am thinking from a personal point of view collaboration is lacking between that board* [Poisons and Medicines Regulatory Authority] *and DAHLD in terms of recommended drugs. The department is responsible to know which drugs to import but with the porous borders anybody can import any drugs into Malawi*. (IDI Government veterinary officer)

To establish a veterinary store, a business permit must be issued from the MTCI. Although an important stakeholder, the DAHLD was not consulted on who was issued a permit, which meant that people without any training in handling veterinary medicines could be issued with a permit. The PMRA and DAHLD had separate mandates which regulated the sale of medicines in the veterinary stores with some overlap between the two. PMRA regulated veterinary shop retail of unexpired medicines, leaving DAHLD to oversee what should be sold in veterinary shops, however these differing mandates lead to gaps in surveillance. Such gaps resulted in some non-recommended medicines being sold to farmers. For example, as noted above we found colistin a drug considered to be of critical importance and banned being sold in the veterinary shops.

### Effects of Political and Economic Liberalisation

Key informants identified two key political events that have shaped the way antibiotics are dispensed in Malawi. Firstly, the implementation of structural adjustment programmes, in the late 1980s which promoted privatisation and reduced size of government departments, thus radically changing the availability of government funding for veterinary officers, and meant they needed to supplement their salaries through private practices. As narrated by one key informant:

*The SAP [structural adjustment] was to wean off the government and farmers from the funding. Moving to a demand led approach, where veterinary officers kept their own drug bags. The veterinary officers now need to be “ready for anything”*. (IDI National level key informant interview)

Further, introduction of multiparty democracy in the early 1990s radically changed government policy, in the veterinary sector it changed the way that farmers interacted with the animal medicine sector. Before the introduction of reforms aimed at opening the economy, the government had the sole responsibility for regulating the handling and distribution of animal medicines including antibiotics. Following the changes, the government opened access to animal medicines and the country witnessed a surge in the number of veterinary stores and private veterinary practitioners. These changes were detailed by one government veterinary officer interviewee who had been working in this field for 34 years:

*In the past veterinary medicines were solely controlled by the government and whenever people wanted animal medicines, they were supposed to go to the government offices to get them, however the coming in of the multi-party Democracy system also led to the privatization of the veterinary services… it removed the burden on the government to be the only one providing such services because in the past it was not possible to go to a shop and buy veterinary medicines… nowadays you would find that a farmer can just go to any veterinary Shop and buy the medicines s/he. We as the government are just supposed to provide Policy direction in terms of veterinary medicines administration to animals.”* (IDI Government veterinary Officer, Blantyre ADD.)

The privatisation of services created a more permissive environment for farmers, allowing them to access a wider range of medicines, often in the absence of affordable veterinary health care services. These broader political and economic decisions continue to shape the ways in which medicines are accessed and used in Malawi.

## Discussion

Current efforts to address AMR have focused on optimising antimicrobial use and reducing dependence on antimicrobials across animal, human and environmental sectors (34). Yet, there are significant gaps in knowledge surrounding antibiotic usage, particularly in Africa (15). Our qualitative study provides an in-depth exploration of antibiotic use in food animals in urban and peri-urban Blantyre, Malawi. Paying particular attention to small-scale intensively farmed animals, one of our most important research findings was the heavy dependence small-scale farmers had on antibiotics. They regularly dosed with antibiotics to prevent and treat disease and ensure growth, and considered the medicines an integral part of the business. In households that kept scavenging and semi-scavenging animals antibiotic use was rare. Farmers and at times shop attendants were unaware that they were dosing their animals with antibiotics through feed and vitamin mixes. Our work speaks to the urgent need to provide farmers, veterinary shops and feed merchants with better information and training on how to safely use antibiotics in feed animals. In Ndirande, where access to space is constrained and at times contested, farmers had little option but to keep animals in cramped spaces within their homes. Farmers also faced challenges in implementing appropriate biosecurity due to infrastructural limitations in their lived environment and their precarious financial position. The poultry market, which was dominated by a small number of powerful producers, made it challenging for farmers to realise sufficient profit to remain solvent, at times leaving them in debt. The cost of veterinary advice and animal antibiotics meant that farmers often under-dosed or slaughtered a sick animal rather than pay for a complete course of antibiotics or engage a veterinary officer. At times farmers killed or sold sick animals, often after animals had been dosed with antibiotics. In this context, antibiotics can be viewed as a form of infrastructure which allowed farmers to keep afloat in a challenging environment (9, 21). Timelier, more affordable access to veterinary services is therefore needed by small-scale intensive farmers to optimise antibiotic use in the context of precarious livelihoods.

Oxytetracycline was the most frequently administered antibiotic, this was often dispensed in mixes with other antibiotics as well as within the feed. This reflects antibiotic use trends both within Africa and worldwide, where the tetracyclines are the most reported antibiotic used in animals (15). We found cotrimoxazole, an antibiotic frequently prescribed in humans was also used to treat animals. This is likely to reflect that cotrimoxazole is cheaper than antibiotics marketed for animals and is one of the most frequently dispensed in primary healthcare in Malawi (35). Regular dosing of animals, which we found evidence of particularly for chickens, exposes the pathogens in animal bodies to subtherapeutic doses, and bacteria are known to be able to develop resistance when exposed to low doses over a long period of time (36). This may also increase exposure to AMR-bacteria to household members (and those in the surrounding community) through air, waste in the environment (36, 37).

Meat was often sold for consumption without adherence to the withdrawal period of antibiotics. The use of antibiotics close to kill is likely to have implications for AMR as it can increase the likelihood of humans becoming exposed to both resistant bacteria and antimicrobial residues through the consumption or handling of contaminated meat (38, 39). In our study, farmers never spoke of withdrawal periods and there was little awareness that giving an antibiotic close to kill was not best practise. Our findings reflected other contexts in Asia and Africa where there was limited knowledge of best practise for antibiotic use to reduce AMR (40–43).

Our findings reflect those from a study in Mozambique found that poultry farming was part of a poverty alleviation strategy for the rural and peri-urban communities (22). In the our study, the extent that farming contributed to household livelihoods, was strongly influential on antibiotic use. In our study antibiotics were rarely used on animals kept for subsistence, which tended to roam freely. This was both because antibiotics were expensive but also because these animals were less likely to get sick.

Our findings that the relative affordability and accessibility of animal and human antibiotics shape which medicines are used and when resonates with those of an ethnographic study in Guatemala, which similarly identified human antibiotics being used with animals (44).

There were marked differences between how medicines including antibiotics were accessed between Chileka and Ndirande. Residents in Chileka had better access to government veterinary officers who were not present in Ndirande. However, in general, farmers in Chileka were poorer and administered fewer antibiotics to their animals. Access to and the availability of veterinary antibiotics was therefore shaped by political, social, and historical events. The government has made various efforts over the past decades to privatise the veterinary profession and make access to medicine easier, but this has resulted in departments and programmes with overlapping mandates. One effect of opening of market gave the government have less control over managing which antibiotics are used by farmers. Further, the reduced funding also prevented the surveillance and enforcement of existing regulations. The subsequent growth in unrestricted access to antibiotics is likely to have important implications for AMR, as a wider range of antibiotics are entering the market.

Although there are many driving factors behind both global and region-specific increases in AMR, antibiotic use in livestock is believed to contribute and some evidence suggests that lowering the use of antibiotics within farming will result in reduced AMR amongst zoonotic bacteria (45). World Health Organization (WHO) guidelines on use of medically important antimicrobials in food-producing animals recommends complete restriction of use of antimicrobials for growth promotion and for disease prevention (45). However, the role that antibiotics play in livestock production, especially for small scale farmers, mean that implementing these guidelines is challenging (46).

We found that information on when and how to treat animals including administering of antibiotics often came through farmers' networks as an important source of information. There is a clear need for provision of more training and access to information about the safer use of antibiotics. This needs to be a whole-sector approach including veterinary shops, veterinary officers, lead farmers and farmers. Even with improved information, however, livelihood precarity also drove both these practices and the unequal market structure in Malawi often left poultry farmers struggling to make sufficient profit to remain support a household. This speaks to the need to address the broader structural drivers of antibiotic use including the unequal market structures that significantly disadvantage small-holder farmers.

Implementation of biosecurity measures to interrupt pathogen transmission and thus improve animal health through optimal hygiene and sanitation, improved shelters away from humans, good animal husbandry practices and timely vaccination of animals are known to be beneficial strategies for reducing the dependency of farmers on antibiotics (45). These could be important interventions to address AMR, particularly in Ndirande where the high density of humans and animals living in close proximity in combination with poor water, sanitation and waste disposal infrastructure increases the risk of environmental transmission (47). Our findings speak to the urgent need to improve infrastructure including water, sanitation, and hygiene to reduce dependency of farmers on antibiotics.

Lastly, we found that food and vitamin mixes containing antibiotics—including those with colistin, a drug considered of critical importance to human health—were being sold by veterinary shops and used by small-scale intensive farmers. These antibiotic mixes were still being manufactured in Europe and exported to Southern Africa. This implies the need for efforts to ensure that regulation in Europe and other countries prevents the manufacture of these mixes for export.

## Limitations

This is one of the first studies to investigate antibiotic use practises in food animals in Malawi and provides important insights into the ways market conditions intersect with social and economic factors to drive antibiotic use. However, the study has several limitations. Firstly, the time the research team spent in veterinary shops was limited to a month. The wide range of brands and vitamins available on the market meant that discussing antibiotic use with farmers could be challenging, as they were not always aware that they were dosing their animals. Using the drug bag when conducing medicine use interviews helped, but some brands may have been missed due to the importation process.

## Conclusion

We found heavy use of antibiotics by small-scale intensive livestock farmers, at times unknowingly in food and vitamin mixes, to ensure that farmers remained solvent. The dependence was shaped by intersecting factors including livelihood precarity, unequal market conditions, timeliness, and affordability of access to veterinary advice and services. The political economy and governance of medicines underpinned these immediate drivers. Our findings speak to the need for a holistic approach to interventions to optimise the use of antibiotics in food animals in Malawi. Interventions to reduce dependence on antibiotics are required. These should include efforts to improve farmers' timely access to affordable veterinary services and provide more information about appropriate antibiotic use including withdrawal periods and feed supplementation. At the policy level efforts are also needed through strengthening of regulation and the enforcement of legislation nationally and globally.

## Data Availability Statement

The raw data supporting the conclusions of this article will be made available by the authors, without undue reservation.

## Ethics Statement

Ethical approval was obtained from the College of Medicine Research Ethics Committee Malawi (COMREC) (P06/182429) and London School of Hygiene and Tropical Medicine Research Ethics Committee (14617). Permission to work in the study communities was provided by the District Health Authorities. Written informed consent or witnessed thumbprint was provided for all participants who were the subject of enquiry for the participant observation, medicine-use interviews, or in-depth interviews. During structured observations in the veterinary stores, customers provided oral consent for their interactions to be recorded anonymously in a notebook.

## Author Contributions

Fieldwork, analysis and drafting of the manuscript was lead by JMa. EM'b, PB, JMu, and HM carried out the fieldwork and contributed to the analysis. EM designed the study and supported the data collection, analysis and drafting of the manuscript. RT and NF provided support with the design and manuscript draft. VS and IC contributed to the analysis and manuscript drafts. All authors contributed to the article and approved the submitted version.

## Funding

This study was funded by AMR Cross-Council Initiative through a grant from the Medical Research Council MR/S004793/1.

## Conflict of Interest

The authors declare that the research was conducted in the absence of any commercial or financial relationships that could be construed as a potential conflict of interest.

## Publisher's Note

All claims expressed in this article are solely those of the authors and do not necessarily represent those of their affiliated organizations, or those of the publisher, the editors and the reviewers. Any product that may be evaluated in this article, or claim that may be made by its manufacturer, is not guaranteed or endorsed by the publisher.
